# Psychophysiological measures of a SuperEnduro athlete during a world championship: an *in-situ* case study

**DOI:** 10.3389/fpsyg.2026.1642621

**Published:** 2026-03-31

**Authors:** Ferenc Ihász, Zoltán Alföldi, Anna Horváth-Pápai, Robert Podstawski, Kevin J. Finn, Mark D. Griffiths, Angéla Somogyi, Attila Szabo

**Affiliations:** 1 Faculty of Health and Sport Science, Széchenyi István University, Győr, Hungary; 2 Doctoral School of Health Sciences, University of Pécs, Pécs, Hungary; 3 Department of Physiotherapy, University of Warmia and Mazury in Olsztyn, Olsztyn, Poland; 4 College of Health, Science, and Technology, University of Central Missouri, Warrensburg, MO, United States; 5 School of Social Sciences, Nottingham Trent University, Nottingham, United Kingdom

**Keywords:** addiction, affect, motorsports, passion, SuperEnduro

## Abstract

**Introduction:**

SuperEnduro is a high-risk motorcycle sport with no prior empirical data. This case study analyzed an elite rider during the fifth round of the 2023/2024 SuperEnduro Grand Prix.

**Methods:**

Psychological measures included core-, positive-, and negative affect, expected and perceived performance, mental and physical exhaustion, perceived risk of racing addiction, and a post-race interview. Physiological measures encompassed heart rate, caloric expenditure, work intensity, and training loads.

**Results:**

Results showed a decline in core affect, with positive affect remaining high and negative affect low after three races. Anxiety decreased progressively, but perceived and objective performance remained low. Elevated physiological measures and subjective perceptions confirmed SuperEnduro’s intensity. The participant self-identified himself as addicted to racing. However, his addiction score was low, suggesting passion or emotional attachment—a blend of pleasure and pain—driving his commitment to race even when injured.

**Discussion:**

These findings provide insight into the physical and psychological demands unique to SuperEnduro athletes.

## 1 Introduction

Motocross is an extreme sport involving high speed, technical skill, and significant physical and cognitive demands (Smith and Mackie, 2016). It requires riders to navigate off-road tracks with jumps, turns, and rough terrain, leading to frequent and severe injuries (Hay et al., 2023; Hino et al., 2017; Grange et al., 2009). The intensity and mental engagement make it an exhilarating experience (Ascensão et al., 2007; Roman et al., 2022), requiring riders to have a high-risk tolerance and excellent physical and mental capacities (D’Artibale et al., 2018; Sharma et al., 2015). However, psychological studies on Motocross are scarce. Since 2000, only a few have examined its mental demands, including two dissertations and studies on anxiety, stress, and psychological performance (Hodkinson, 2022; Patel, 2006; De Mojà and De Mojà, 1986; Lehmann et al., 1982; Collins et al., 1993). Recent research by Komáromi et al. (2024) found that post-race arousal, anxiety, and positive affect decreased, with performance evaluations linked to emotional states. Core affect changes were visualized using a valence-activation grid (Russell, 2003; Kovácsik and Szabo, 2019).

SuperEnduro, a distinct form of off-road motorcycle racing, differs from Motocross in its indoor, obstacle-heavy, and shorter-track format. It merges elements of Enduro, Motocross, and road racing, requiring constant adaptation and decision-making. Races are held in categories (Prestige, Junior, Women) and demand high physical and mental resilience. Despite growing popularity, e.g., 13,000 spectators at the 2024 Hungarian GP, research on SuperEnduro is limited. Nomothetic studies are impractical due to sport’s intensity and risks; hence, this study adopted an idiographic approach.

We investigated a rider, “Oliver,” during the 2024 SuperEnduro World Championship in Budapest. Despite a knee injury from prior races, Oliver competed in the Prestige category, risking re-injury, and participated in our study. He wore a Polar Team Pro Monitor for physiological data and completed pre- and post-race psychological assessments. A follow-up interview helped us interpret his data.

Oliver, 28, has been riding since age five and has won 12 championships in Motocross, Enduro, and Enduro Cross. He qualified for the Prestige category after medaling in the 2023 European Cup. The study applied the Transactional Theory of Stress and Coping (TTSC) (Lazarus and Folkman, 1987) to explore how Oliver perceived and managed the stress of racing. TTSC posits that individuals appraise situations as threats or challenges and use coping strategies accordingly. Oliver’s emotional and physiological data were interpreted considering these appraisals and coping styles. Finally, we examined symptoms of behavioral addiction (Hardin and Higgins, 1996; Griffiths, 2005), including salience, conflict, mood modification, and withdrawal, to assess his relationship with the sport.

## 2 Materials and methods

### 2.1 Setting and background

The sport is essentially a combination of enduro, motocross, and trial racing. There are different sections, such as enduro (Hay et al., 2023), typically with wooden and stone obstacles, motocross elements (Hino et al., 2017), such as jumps, wave rows, mandiners, and turns, and trial skill (Grange et al., 2009) elements with higher structures. The fifth round of FIM’s 2024 SuperEnduro World Championship took place at Budapest’s MVM Dome on February 3, 2024. The timeline diagram shows the nature of the injury. The FIM SuperEnduro World Championship is an exciting motorsport held in indoor arenas around the world. The track consists of soil (covered and compacted earth) and artificial obstacles. The obstacles include: rocks, wooden beams, concrete pipes, logs of various diameters, earth jumps, large tires with a diameter of 2 meters, etc.

The courses are 250–300 meters long. The appearance of the course, its degree of difficulty, and the obstacles placed on it are entirely up to the local organizer, which means that competitors do not know what the course will look like until the last moment. This provides great freedom, but at the same time makes the task very difficult.

The competition day begins with morning practice sessions, where entrants must qualify for the evening races. Fourteen drivers in each category can advance to the finals. Those who fail to qualify directly still have a chance in the consolation round, known as the “Last Chance” race, where the top two finishers advance.

Important rules: Competitors qualify for the final during time trials. Competitors may not receive outside assistance on the track, except from track marshals performing their duties for safety reasons. Competitors may only use the track. However, if they accidentally leave the track, they may safely return to the track from the point closest to where they left the track without gaining an advantage. Leaving the track is prohibited. Attempts to gain an advantage by leaving the track may result in a 5-s time penalty or disqualification, at the discretion of the FIM judge. The difficulty of the track makes this race extremely exciting for spectators. Here, it is not speed that is most important, but the combined work of the motorcycle and the rider. Despite a recent injury, Oliver competed in the Prestige category for the first time, finishing the race while risking re-injury. He also participated in this study, wearing a Polar Team Pro Monitor to track physiological data and complete psychological assessments before and after each race. To better interpret the data, a follow-up phone interview was conducted the next day to explore his motivations and reactions to both subjective and objective measures.

### 2.2 The participant

For Oliver, who was 28 years old at the time of the study, sport is a part of his family’s everyday life. While he devotes his life to motorsport, his brother competes at national and international levels in wrestling. He has been riding motorcycles since the age of five. Throughout his career, he has won 12 championship titles, numerous cups and medals in Motocross, enduro, and enduro cross disciplines, becoming a medalist in the 2023 SuperEnduro GP Europe Cup, which meant he qualified for participation in the ‘Prestige’ category in 2024. Less than one month before this study, Oliver suffered two injuries during the third and fourth rounds of the SuperEnduro World Championship. Despite his knee injury, he insisted on racing and agreed to participate in this study conducted during the fifth round of the 2024 SuperEnduro World Championship in Budapest, Hungary.

### 2.3 Measures and instruments

#### 2.3.1 Psychological measures

**Figure 1:**
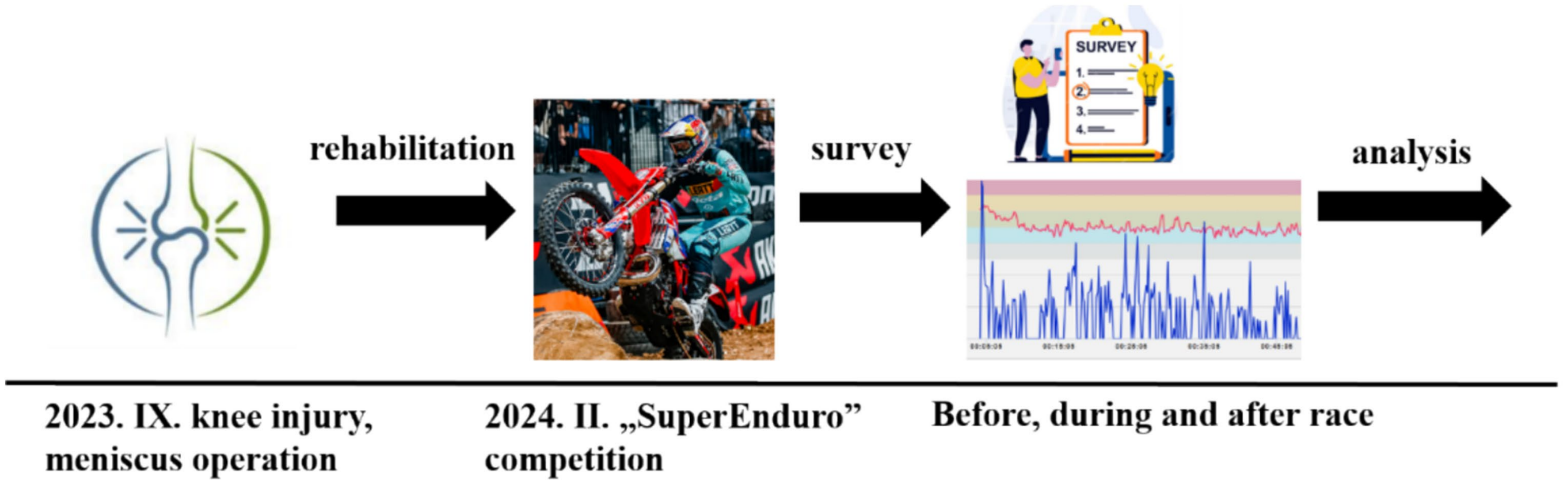
Study flow diagram.

Affective valence was measured before and after each of the three races using the Feeling Scale (FS; 19), which ranges from −5 (‘very bad’) to +5 (‘very good’). Activation was assessed with the Felt Arousal Scale (FAS; 20), a 6-point Likert scale from 1 (very low) to 6 (very high). Both scales are psychometrically sound (Hardy and Rejeski, 1989; Svebak and Murgatroyd, 1985). Their combined values were plotted on an affective grid (Figure 1) to represent core affect, based on Russell’s model (Russell, 2003) and the design used by Komáromi et al. (2024) for comparability with Motocross data.

The 10-item Positive and Negative Affect Schedule (PANAS; Gyollai et al., 2011), derived from Watson et al. (1988), was also used pre- and post-race to assess reflective emotional states. It includes five positive (e.g., alert, determined) and five negative (e.g., nervous, jittery) affective items rated on a 5-point scale. Subscale scores yield separate positive affect (PA) and negative affect (NA) scores.

State anxiety was assessed pre-race using a validated single-item 10-point scale (Williams and Smith, 2013), known for high sensitivity (71–87%) and specificity (73–88%) (Williams and Smith, 2013), and shown to be reliable under time constraints (Davey et al., 2007). Expected and perceived performance were measured before and after each race with two questions rated from 1 (very poor) to 10 (very good).

Post-race, Oliver rated perceived physical and mental exertion using the 10-point Borg scale, which is the rate of perceived exertion (Borg, 1982). The day after, he completed the Exercise Addiction Inventory (EAI; Szabo et al., 2019; Szabó, 2021), modified by replacing “exercise” with “SuperEnduro.” Items such as “SuperEnduro is the most important part of my life” assessed addiction symptoms (salience, conflict, mood modification, tolerance, relapse, withdrawal) based on the components model of addiction (Griffiths, 2005). Responses on the 6-point Likert scale yield a total score, with 29+ indicating addiction risk. The scale has good internal consistency (Cronbach’s *α* = 0.71; 31).

The day after the race, Oliver also answered a few questions via telephone regarding his motive for racing while being injured, his thoughts about the race, his psychological and physiological measures rating, and their connection. These questions framed around the TTSC were necessary to ensure that the researchers (Jevon and O’Donovan, 2000); interpretation of the data matched Oliver’s interpretation. After completing the EAI, Oliver was asked the following questions within a short 10-min interview:

1. What was your main reason for participating in this Grand Prix?
2. How did you feel about competing in the Hungarian Grand Prix?
3. What were the physical and mental challenges you encountered?
4. How did the spectators and the injury affect your performance?
5. What was your most significant fear during the race?
6. How did your anxiety change throughout the race?
7. How was your performance in this Grand Prix?

#### 2.3.2 Physiological measures

Physiological measures were recorded using a bani Team Pro monitor (Polar Electro Co., Kempele, Finland) and analyzed with Polar Team Pro software, version 2.0. The system includes a chest belt with a Polar H7 Bluetooth 4.0 sensor, built-in ECG electrodes, a 10 Hz GPS, and a 200 Hz micro-electromechanical motion sensor. After each session, all wearable sensors are docked for data synchronization to an iPad, with the data transferred wirelessly to the Polar Team Pro web service via Wi-Fi or cellular connection. The system generates data on heart rate (HR), location, distance, velocity, acceleration, and power. It also uses a built-in algorithm to calculate the Training Impulse (TRIMP). The Bluetooth-enabled sensors allow reliable and continuous data transfer within a range of up to 200 meters (Polar Research Center, 2022). This setup was used to measure HR, race load (TRIMP based on intensity in watts), race duration, and energy expenditure (Kcal), providing an overall assessment of training stress. Exercise physiologists widely use the TRIMP to quantify an exercise session’s physiological load or intensity. It was introduced by Banister (1991) to assess the cumulative stress placed on an athlete’s body during training. TRIMP considers both the duration and intensity of a workout, often using HR as the critical indicator of intensity. It is calculated using an algorithm built into the Polar monitor. TRIMP calculations are typically based on how much time an athlete spends in different HR zones, which reflect varying levels of exercise intensity, and time spent in each HR zone multiplied by an intensity factor. More time in higher HR zones (closer to maximum HR) contributes to the overall TRIMP score, which measures the training load.

#### 2.3.3 Motorcycle

In all three races, Oliver rode a RIEJU MR 300cc (2024) motorcycle at the Hungarian Grand Prix. The RIEJU MR 300cc (2024) manufactured in Figueres, Spain, is a high-performance enduro motorcycle for off-road riding enthusiasts. Powered by a 300cc, two-stroke engine, it offers a powerful yet manageable ride, ideal for navigating rugged terrains. It features advanced suspension systems, including Kayaba forks, a lightweight chassis, and a modern fuel injection system. This ensures precise handling, optimal power delivery, and enhanced durability for competitive off-road use. The bike also comes with high-quality components like Nissin brakes, Renthal bars, and adjustable settings, making it versatile for various rider preferences.

### 2.4 Procedure

On race day, the research protocol was explained to Oliver, and he was instructed to act naturally as if not participating in the study. One hour before the first race, Oliver was fitted with the Polar Team Pro chest heart rate monitor in the race pit area, which remained on until approximately one hour after the final race, with continuous recording throughout this period. While waiting before the race, Oliver sat in the designated pit area and focused on the upcoming race. The physiological data from the monitor were followed in real time on an iPad by the examiner. These data were automatically stored by the system and were available for immediate analysis.

Five minutes before lining up for each race, Oliver completed the state psychological measures, which took approximately one minute. After each race, once he had settled at the finish area, he completed the post-race questionnaires, which were identical to the pre-race ones except for the questions assessing perceived performance and mental and physical effort. Two researchers were present: one read the questions aloud while the other recorded the responses (Figure 1).

### 2.5 Data extraction

Physiological recordings were extracted as 30-min averages for the anticipatory and recovery periods before and after the races, respectively. The recordings during the three races were isolated with time stamps and averaged for each race period, lasting between seven and 8 minutes (Table 1).

**Table 1:** Physiological measures before, during, and after three races.

Measure	Race number	Before (M of 30 min)	During (M ≈ 7.25 min)	After (M of 30 min)
Mean heart rate (beats per minute)	1	125	173	126
	2	129	169	132
	3	130	170	115
Maximum heart rate (beats per minute) ^c^	1	159	188	152
	2	149	185	149
	3	148	184	133
Watts (mean)	1	194	304	58
	2	102	302	81
	3	190	298	70
Watts (maximum) ^c^	1	520	1,234	196
	2	480	969	209
	3	205	987	196
Kilocalories (Kcal)	1	347	220	350
	2	251	229	348
	3	339	221	119
Race load (Training Impulse [TRIMP])^a^	1	32	34	31
	2	22	34	32
	3	32	33	9
Race time, placement, and the number of laps completed^b^	1	7:39:882	10th place	9 laps
	2	7:16:418	11th place	9 laps
	3	7:19:592	11th place	9 laps

a This is a commonly used arbitrary unit that the Polar measuring instrument calculates. Higher values reflect higher physical efforts.

b Data retrieved May 09, 2024, from: https://enduro21.com/en/racing/latest/superenduro/2024-superenduro-results-billy-bolt-at-his-best-in-budapest.

c These values are not means, but the highest value registered in the given period.

M = mean value.

## 3 Results

### 3.1 Psychological (subjective) measures

**Figure 2:**
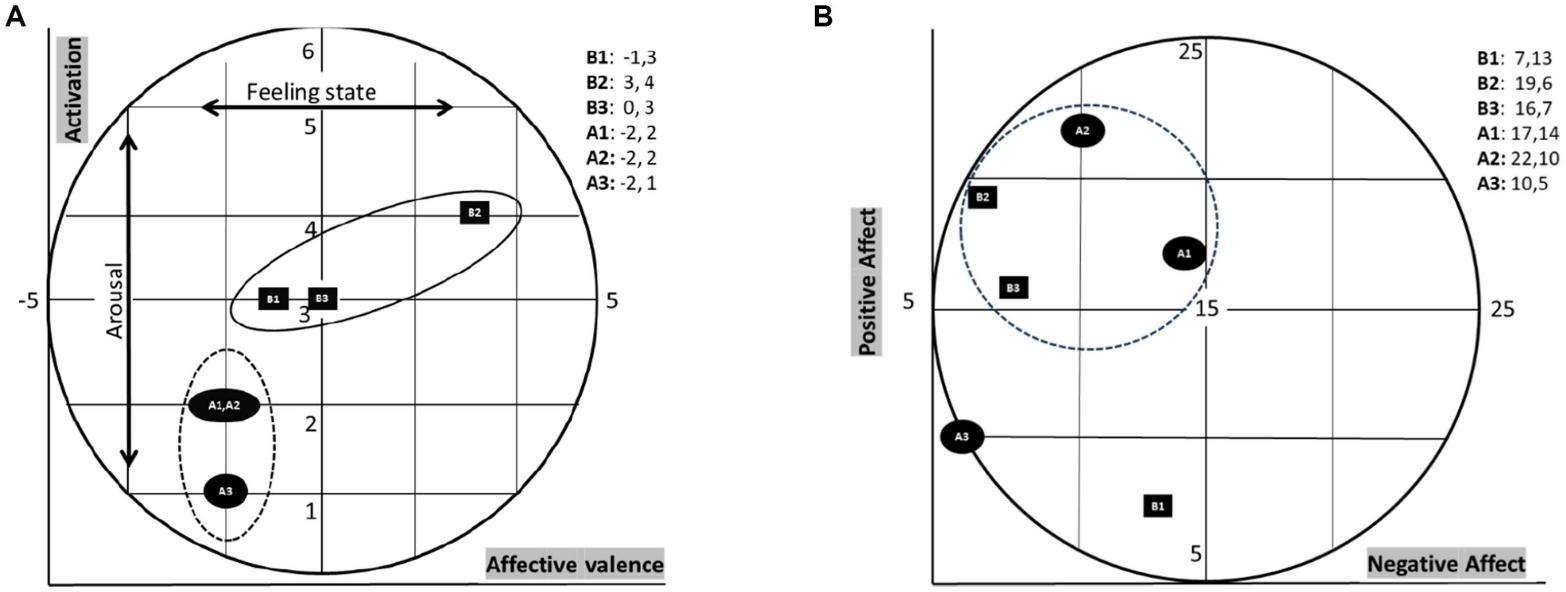
**(A)** Oliver’s core affect before and after three races. The letter B stands for “before,” and the letter A stands for after. The numbers after the letters identify the race number. In the second race, the competitors started the race in reverse order of qualification. **(B)** Oliver’s positive and negative affect junctions (i.e., B1 positive affect = 7, negative affect = 13) before and after three races. The letter “B” stands for “before,” while the letter “A” stands for “after.” The numbers after the letters identify the race number. Competitors started the second race in reverse order of qualifying. The inner broken circle illustrates that, apart from before the first and after the last race, Oliver’s affective states were in the high positive–low negative affect quadrant.

**Figure 3:**
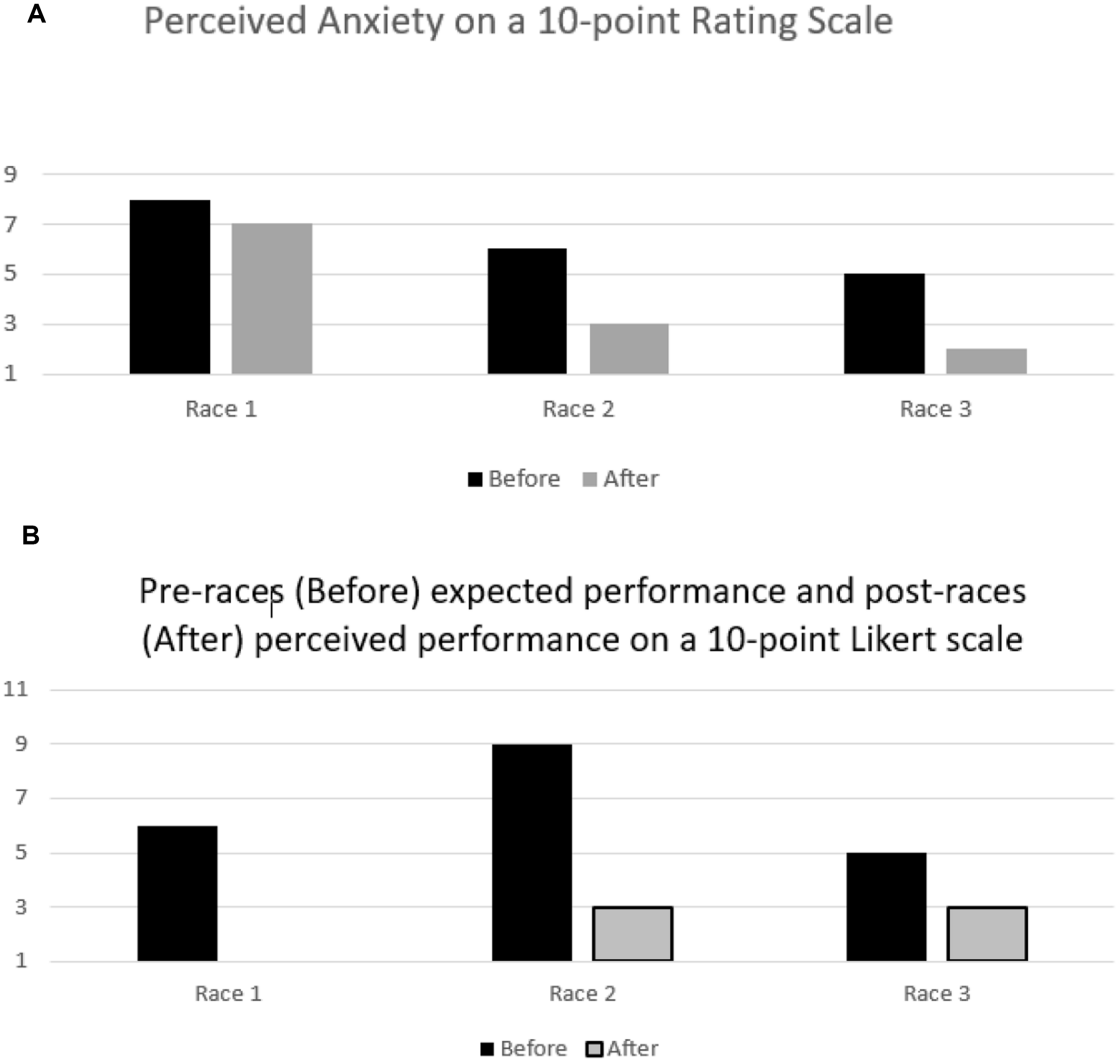
**(A)** Oliver’s perceived anxiety was lower after the first race and after each race. In race two, the start line was in the reverse order of the qualification. **(B)** Oliver’s expected vs. perceived performance. After race 1, the perceived performance was right on the minimum (worse possible) score = 1, so no bar is seen in the figure after race 1.

Oliver found the races very exhausting mentally and physically, based on the 10- item Borg scale, on which he scored 9.33/10 on mental exhaustion and 10/10 on physical exhaustion across the three races. Oliver’s core affect [momentary non-reflective feeling state] was, in all instances, higher before than after the race (Figure 2A). His positive and negative affect, apart from before the first race and after the last race, were in the high positive to low NA quadrant (Figure 2B). His anxiety was lower after each race (Figure 3A). Moreover, his perceived performance was always lower than what he expected (Figure 3B). Finally, his score on the adapted EAI-R was 25/36, lower than the cutoff score of 29/36 (Szabó, 2021), which would qualify him as being at risk of addiction to the sport.

### 3.2 Physiological (objective) measures

All physiological measures were higher compared to the 30 min preceding it and the same period after it. However, the average mean values observed pre- and post-race (Table 1) were still high because they reflected anticipatory (before the race) and recovery (after the race) periods. Oliver’s average resting HR was 55 beats per minute (bpm), as retrieved from his smartwatch. Therefore, using this input value, based on the Karvonen formula (She et al., 2014), Oliver’s HR ranged between 88 and 90% of his maximal HR in the three races (Table 1).

Before and after the races, Oliver’s HR fluctuated between 60 and 69% of his maximum HR, corresponding to moderate resistance and aerobic training load (Da Silva et al., 2020). His mean physical effort exerted during the races was around 300 watts, reflecting a high intensity. Oliver burned an average of 223.33 kilocalories (Kcal) during the average ≈7.25-min races (Table 1). His training (race) load, expressed as training impulse (TRIMP) calculated by a built-in algorithm of the Polar monitor from HR and race duration (Banister, 1991), was (Willegers, 2022; Deroche et al., 2011) in the three races.

### 3.3 Post-race interview

This section summarizes Oliver’s answers to the seven questions asked in the brief interview (see Methods section). Oliver described his involvement in SuperEnduro as an “addiction,” expressing eagerness to compete in the more challenging Prestige category despite the risk of injury and potentially lesser recognition than in the junior category (Q1). He felt excited and proud competing in front of enthusiastic spectators, with this excitement evident the day after the race (Q2). Oliver found the race extremely tiring, particularly in the second half, compared it to gladiator fights, and struggled with his knee injury (Q3). Racing in front of fans motivated him, but he worried that his performance might have been negatively judged due to his injury, even though spectators were unaware of it (Q4). His greatest fear was death, which was most prominent before the race but dissipated once he entered a focused state. He feared that any distraction from this state could lead to severe injury or even death (Q5). Oliver perceived his anxiety decreasing after each race due to reduced stress and challenge (Q6). He rated his performance as low due to his knee injury and inability to enter a flow state, which affected his satisfaction. However, finishing the race was still a positive experience for him (Q7).

This section may be divided by subheadings. It should provide a concise and precise description of the experimental results, their interpretation, as well as the experimental conclusions that can be drawn.

## 4 Discussion

This study is the first to explore the psychophysiological responses of a SuperEnduro rider in a world championship setting and the first to examine the sport academically, despite its 25-year history. Though a single-case study, it offers a foundation for future research and illustrates sport’s intense mental and physical demands. Oliver’s Borg scale ratings for psychological and physical effort surpassed those reported by Komáromi et al. (2024) in Motocross, suggesting that SuperEnduro may be more demanding; however, his injury might have influenced these ratings.

Oliver burned an average of 223.33 Kcal in ≈7.25-min races (1,848 Kcal/h), over five times the estimated hourly expenditure in Motocross (236–345 Kcal), aligning with his Borg rating of 10 for race intensity. His training load (TRIMP) was high and consistent, with TRIMP/min values exceeding 4.5, well above those reported in sports like hockey or soccer.

Other physiological markers (watts and HR) confirmed the races and related periods were strenuous. His 300-watt output matched peak levels in incremental exercise research. HR ranged between 60 and 69% of maximum during anticipation and recovery (moderate aerobic effort), while peak HR during races reached 184–188 bpm—comparable to values in much younger Motocross athletes. Such heart rate levels are typical in motorsports, where they often exceed 90% of the maximum heart rate. In many cases, riders exceed the anaerobic threshold, which places a significant physiological and mental strain on them (Ihász et al., 2024).

In a case study of an injured elite athlete, interventions combining relaxation techniques, imagery, positive self-talk and affirmations, goal setting, negative thought stopping, and cognitive restructuring were found to be successful in treating addiction and commitment (Willegers, 2022).

Despite high HRs, post-race arousal and anxiety were low, possibly due to psychological relief or disappointment. This dissociation aligns with prior research on high-risk athletes. Unlike Motocross riders, whose core affect post-race showed high arousal and positive valence, Oliver’s core affect only reached that zone before his second race, possibly due to his injury.

Contests of this type like SuperEnduro, and the heart rate pattern analysis performed there, provide excellent information for coaches, before, during, and after the preparation period.

### 4.1 Post-race interview

In the post-race interview, Oliver described his motivation as an “addiction,” likely reflecting emotional attachment or passion, though passion was not formally assessed. His attachment may also serve as an emotional regulator. The crowd energized him, supporting social facilitation theory, while concerns about performance evaluation may have lowered post-race affect, consistent with shared reality and spontaneous representation theories (Deroche et al., 2011).

Fear of injury and death stemmed from both the sport and his reduced control due to injury, aligning with theories linking lower perceived control to increased fear in high-risk sports. Although Oliver reported lower post-race anxiety, this was not supported by HR data, highlighting a known disconnect between physiological and subjective responses. He rated race performance low but appreciated finishing, with slight increases in reflective positive affect after the first two races.

### 4.2 Limitations

This case study reflects the experience of one injured athlete and is not generalizable. Potential Hawthorne effects may have influenced subjective measures. Injury likely affected both psychological and physiological responses. Activities during anticipation and recovery were not assessed, which may have missed potential insights. Though the EAI showed no risk of addiction, passion was not measured, limiting the interpretation of his “addiction” claim. Contextual race variables (setting, spectators, qualification) may have also influenced results (Szabo and Demetrovics, 2022).

## 5 Conclusion

Despite its limitations, this study suggests that SuperEnduro is a high-intensity sport, supported by both subjective and objective indicators. Underperformance was accompanied by decreased non-reflective core affect, while reflective positive affect remained high, likely due to satisfaction with completing the race without re-injury. Lower post-race anxiety and arousal may indicate mental recovery and acceptance of performance limitations, as supported by interview data. Despite injury and subpar performance, Oliver enjoyed the challenge, and his self-perceived addiction likely reflects strong passion rather than true exercise addiction, as his EAI score did not indicate risk. Further research should explore passion in high-risk sports to confirm this interpretation. While *in situ* studies in extreme sports are difficult, additional research is encouraged. This study highlights the considerable physical and mental demands of SuperEnduro. Coaches and athletes should consider these factors when designing training programs that support both physical readiness and psychological resilience for safe, effective performance.

## Data Availability

The datasets presented in this article are not readily available because the dataset is not publicly available due to participant privacy and ethical restrictions, but it can be provided by the authors upon reasonable request. Requests to access the datasets should be directed to Ferenc Ihász, ihasz.ferenc@sze.hu.
